# Catheter ablation of atrial fibrillation in patients with heart failure - preserved and reduced ejection fraction - real world evidence

**DOI:** 10.1007/s10840-026-02349-z

**Published:** 2026-05-26

**Authors:** Maximilian Moersdorf, Qussay Marashly, Christian Massad, Yara Menassa, Han Feng, Michel Abou-Khalil, Mohammad Montaser Atasi, Alex El Darzi, Carlo El Khoury, Philipp Sommer, Amitabh C Pandey, Christian Sohns, Nassir Marrouche

**Affiliations:** 1 Tulane Research Innovation for Arrhythmia Discovery, New Orleans, LA USA; 2 Department of Electrophysiology, Heart and Diabetes Center NRW, Ruhr University Bochum, Bad Oeynhausen, Germany; 3 Tulane University School of Medicine, 1430 Tulane Avenue, New Orleans, LA 40130 USA

**Keywords:** Atrial fibrillation, Heart failure, Catheter ablation, HFpEF, HFrEF, All-cause mortality

## Abstract

**Background:**

Catheter ablation for atrial fibrillation (AF) has been shown to reduce mortality and morbidity in patients with AF and heart failure with reduced ejection fraction (HFrEF) compared with medical therapy (GDMT) alone. Outcomes in patients with preserved ejection fraction remain less clearly defined. This study compared long-term outcomes of catheter ablation in patients with AF and heart failure (HF) across the spectrum of ejection fraction using real-world data.

**Methods:**

This retrospective cohort study utilized the U.S. Collaborative Network in TriNetX to identify adults with AF and HF between September 2018 and September 2024. After 1:1 propensity score matching, 12,492 patients with HFpEF and AF and 38,150 patients with HFrEF were included. The primary endpoint was a composite including all-cause mortality, left-ventricular-assist device implantation, or heart transplantation. Secondary endpoints included hospitalization, stroke, and antiarrhythmic drug (AAD) use. Outcomes were compared between patients receiving ablation and GDMT versus GDMT alone.

**Results:**

Over a mean follow-up of 973 days (HFpEF) and 926 days (HFrEF), AF ablation was associated with lower risks of the primary endpoint and mortality (HFpEF: hazard ratio (HR) 0.29; HFrEF: HR 0.40; all *p* < 0.001), as well as reduced hospitalization (HFpEF: HR 0.81; HFrEF: HR 0.75; all *p* < 0.001) and stroke (HFpEF: HR 0.86, *p* = 0.025; HFrEF: HR 0.76, *p* < 0.001). Patients that received ablation had more frequent AAD use despite greater cardioversion rates (HFpEF: HR 1.37; HFrEF: HR 1.08, all *p* < 0.001).

**Conclusion:**

Catheter ablation conferred comparable reductions in mortality, advanced HF therapies, stroke, and hospitalization in both HFpEF and HFrEF patients.

**Graphical abstract:**

**Figure:**
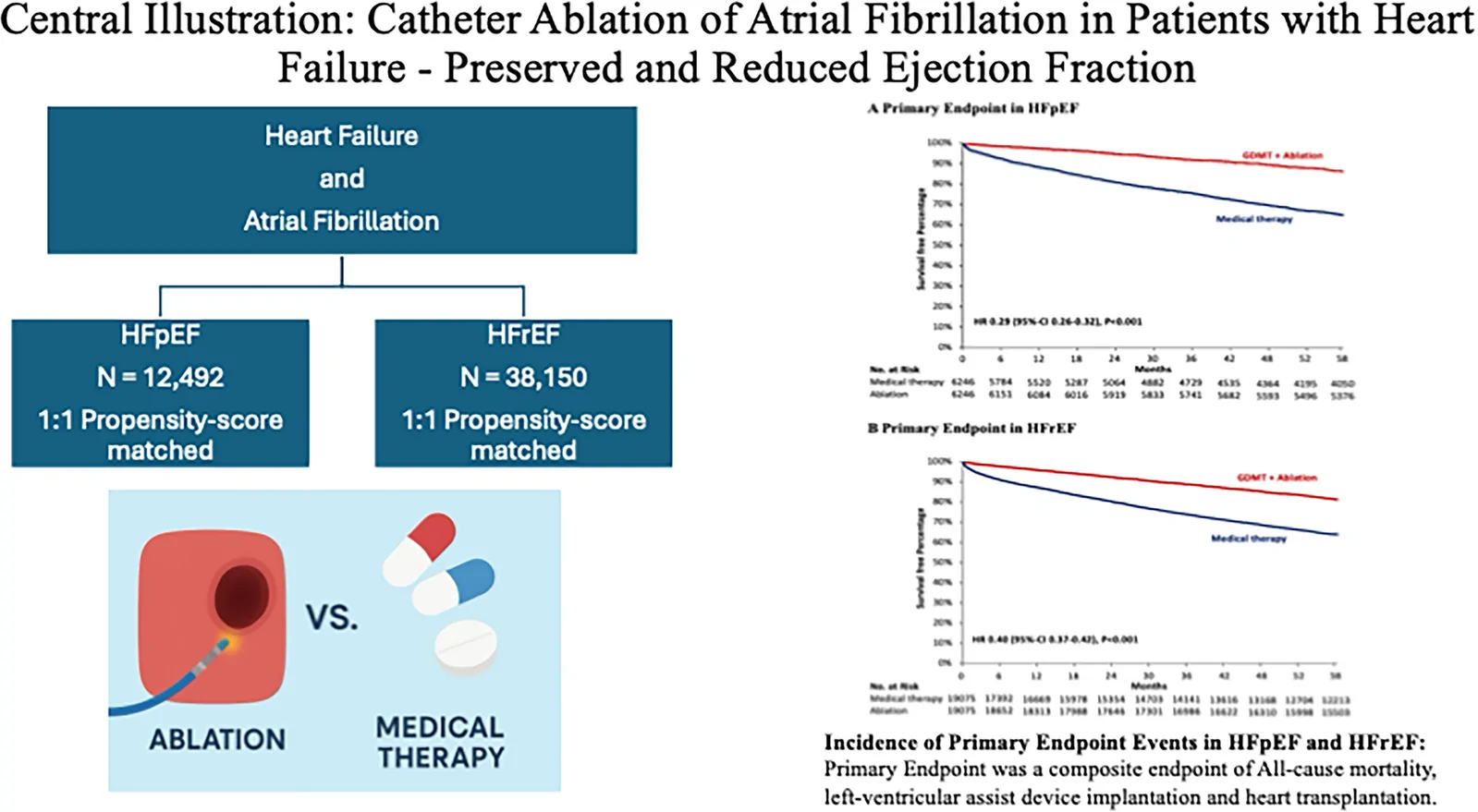

**Supplementary Information:**

The online version contains supplementary material available at https://doi.org/10.1007/s10840-026-02349-z.

## Introduction

Catheter ablation for atrial fibrillation (AF) has shown to significantly lower mortality and morbidity among patients with AF and heart failure (HF) compared with medical therapy alone [1–3]. The CASTLE-AF trial established the survival and HF–related benefits of AF ablation, including reduced mortality, fewer hospitalizations, and delayed HF progression [1]. More recently, the CASTLE-HTx trial extended these findings to patients with end-stage HF [2]. Although the benefit of ablation in patients with HF with reduced ejection fraction (HFrEF) is well recognized, outcomes in patients with HF with preserved ejection fraction (HFpEF) remain less clearly defined. A subanalysis of the CABANA trial suggested that maintenance of sinus rhythm in patients with AF may attenuate HF progression and reduce cardiovascular hospitalizations in the HFpEF population [4].

This study aimed to compare the long-term outcomes after catheter ablation among patients with AF and HF across the spectrum of ejection fraction, specifically contrasting those with HFpEF and HFrEF, using real-world data to address gaps in existing evidence.

## Methods

### Study design

This study utilized data from TriNetX, a global clinical research network that aggregates de-identified electronic medical records data from participating health care organizations [5]. The TriNetX database integrates data including patients’ demographics, diagnoses, procedures, medications, and laboratory results.

All data provided through TriNetX network are de-identified and aggregated, ensuring compliance with the Health Insurance Portability and Accountability Act and other applicable US government regulations. Further information about the TriNetX network is publicly available at https://trinetx.com/data-sets-analytics/.

Patient demographics and diagnoses were obtained according to the International Classification of Diseases (ICD), ninth and tenth versions (ICD-9 and ICD-10). Data on procedures, laboratory tests, and medications were also obtained using the TriNetX network.

### Patient population

Using the TriNetX database, we identified adult patients with coexisting AF and HF, including HFpEF or HFrEF, between September 1, 2018, and September 1, 2024. AF was defined by ICD codes I48.0 or I48.1 or I48.2 or I48.91. HFpEF was defined by ICD codes: I50.3. HFrEF was defined by ICD codes: I50.2 or I50.4. Due to the absence of a specific ICD code for HF with mildly reduced ejection fraction (HFmrEF) and the inconsistent availability of left ventricular ejection fraction measurements for cohort classification within the TriNetX network, the HFrEF cohort identified by ICD codes may also encompass patients with HFmrEF. Patients were then stratified according to whether they underwent catheter ablation for AF, identified using SNOMED code 233,159,005 and CPT codes 93,656 and 93,657.

Patients with a prior diagnosis of HFrEF were excluded from the HFpEF cohort. A complete list of all ICD-10-CM codes used in this analysis is provided in Supplemental Table 1. Data for the ablation cohort were provided by 53 and 49 participating health care organizations in the HFpEF and HFrEF groups, respectively. Data for the non-ablation cohort were provided by 80 and 81 health care organizations in the HFpEF and HFrEF groups, respectively. The index date was defined as the date of the first documented AF diagnosis or catheter ablation procedure, with follow-up beginning 1 day later and continuing until the occurrence of an outcome or a maximum follow-up of 5 years.

### Patient outcomes

The primary outcome was a composite of all-cause mortality, left-ventricular assist device implantation, or heart transplantation during the 5-year follow-up period, as identified using ICD-coded diagnoses.

Secondary outcomes including all-cause hospitalization, stroke, antiarrhythmic drug (AAD) use, cardioversion procedures, and all-cause mortality.

### Statistical analysis

All statistical analyses were conducted using the TriNetX online research platform. Chi-square analysis and independent sample t-tests were used to compare categorical variables and continuous variables, respectively. Patients were propensity score matched in a 1:1 ratio based on the following variables: age at index, sex, diabetes mellitus, cardiomyopathy (dilated or ischemic), ischemic heart disease, cerebral infarction, chronic obstructive pulmonary disease, hypertension, AF type (paroxysmal/persistent/chronic atrial fibrillation), atrial flutter type (typical or atypical), diastolic (congestive) heart failure, presence of cardiac pacemaker, presence of automatic (implantable) cardiac defibrillator, medication (cardiovascular (beta blocker, ACE inhibitor, angiotensin II inhibitor, spironolactone, sacubitril), antiarrhythmics (class I and III, class III, sotalol, amiodarone, flecainide), digitalis glycosides, diuretics, anticoagulants, warfarin, sodium-glucose co-transporter 2 (SGLT2) inhibitors), laboratory parameters (creatinine, glomerular filtration rate, NT-pro BNP), as well as left ventricular ejection fraction (LVEF), heart rate, body mass index, and body surface area. A complete list of matching variables is provided in Supplementary Table 2.

Cox regression analysis was used to calculate the hazard ratios (HRs) and confidence intervals (CIs) for the primary and secondary outcomes.

## Results

### Baseline patient characteristics

Before propensity score matching, a total of 349,815 patients with coexisting AF and HFpEF and 577,460 patients with AF and HFrEF were identified. Of these, 9,153 patients in the HFpEF group and 22,481 in the HFrEF group underwent catheter ablation. Patients in the ablation cohorts were significantly younger at the index event (HFpEF: 70.4 vs. 76.6; HFrEF: 66.5 vs. 72.9, *p* < 0.001) and more likely to be male in the HFrEF cohort (71.8% vs. 62.4%, *p* < 0.001). Additional baseline characteristics before propensity score matching are provided in Supplementary Table 3.

After propensity score matching, the matched cohorts included 12,492 patients with HFpEF and 38,150 patients with HFrEF, evenly divided into ablation and non-ablation groups (6,246 and 19,075 patients per group, respectively) (Supplementary Table 4). The female proportion in the HFpEF cohort after propensity score matching remained higher with 53.1% compared to 30.1% in the HFrEF cohort. The compressed baseline characteristics of the propensity score matched cohorts are presented in Table 1.

**Table 1:** Baseline characteristics after propensity score matching

	**HFpEF**			
**Characteristic**	**Ablation**	**Medical therapy**	***P*-value**	**Standard difference**
	**(*n* = 6246)**	**(*n* = 6246)**		
Age at index — yr	71 ± 9	71 ± 12	0.022	0.041
Male sex — no. (%)	2933 (47)	2911 (47)	0.693	0.007
Body-mass index	33 ± 7	33 ± 9	0.126	0.027
Body surface area	2 ± .4	2 ± .3	0.134	0.027
Left Ventricular Ejection Fraction (LVEF)				
Available — no. (%)	1652 (26)	1584 (25)	0.160	0.025
LVEF (%)	60 ± 8	60 ± 12	0.393	0.030
LVEF ≥ 40 to 50% — no. (%)	31 (.5)	37 (.6)	0.470	0.013
LVEF < 40% — no. (%)	0 (.0)	0 (.0)	1.000	0.000
Persistent atrial fibrillation — no. (%)	3298 (53)	3335 (53)	0.507	0.012
Typical atrial flutter — no. (%)	794 (13)	817 (13)	0.539	0.011
Atypical atrial flutter — no. (%)	511 (8)	510 (8)	0.974	0.001
Heart rate — bpm	75 ± 19	74 ± 16	0.509	0.012
Cardiomyopathy — no. (%)	858 (14)	830 (13)	0.464	0.013
Dilated cardiomyopathy — no. (%)	116 (2)	99 (2)	0.242	0.021
Ischemic cardiomyopathy — no. (%)	104 (2)	115 (2)	0.453	0.013
Ischemic heart disease — no. (%)	3483 (56)	3439 (55)	0.428	0.014
Hypertensive diseases — no. (%)	5500 (88)	5374 (86)	0.001	0.060
COPD — no. (%)	1384 (22)	1304 (21)	0.082	0.031
Diabetes mellitus — no. (%)	2304 (37)	2261 (36)	0.424	0.014
Cerebral infarction — no. (%)	546 (9)	520 (8)	0.405	0.015
Presence of cardiac pacemaker — no. (%)	575 (9)	535 (9)	0.208	0.023
Presence of ICD — no. (%)	151 (2)	141 (2)	0.554	0.011
NT-pro BNP available — no. (%)	2160 (35)	2053 (33)	0.045	0.036
NT-pro BNP — pg/ml	2140 ± 4941	2766 ± 5623	0.001	0.118
Creatinine — mg/dl	1 ± 1	1 ± 3	0.038	0.037
Glomerular filtration rate (MDRD) — ml/min	64 ± 22	63 ± 28	0.030	0.039
Medication — no. (%)				
Beta blocker	5414 (87)	5352 (86)	0.108	0.029
Diuretics	4933 (79)	4888 (78)	0.326	0.018
RAS-inhibitor	4152 (66)	4042 (65)	0.041	0.037
ACE-inhibitor	1963 (31)	1903 (30)	0.246	0.021
Angiotensin II inhibitor	2189 (35)	2139 (34)	0.347	0.017
Sacubitril - valsartan	84 (1)	75 (1)	0.473	0.013
Spironolactone	1002 (16)	1021 (16)	0.644	0.008
SGLT-2 inhibitor	510 (8)	493 (8)	0.576	0.010
Antiarrhythmics	5194 (83)	5161 (83)	0.433	0.014
Class I and III	5179 (83)	5149 (82)	0.478	0.013
Class III	2818 (45)	2927 (47)	0.050	0.035
Amiodarone	2225 (36)	2329 (37)	0.053	0.035
Digitalis glycosides	811 (13)	831 (13)	0.596	0.009
Anticoagulants	5728 (92)	5691 (91)	0.237	0.021
Warfarin	1082 (17)	1082 (17)	1.000	0.000
**Characteristic**	**HFrEF Ablation**	**Medical therapy**	***P*-value**	**Standard difference**
	**(*n* = 19075)**	**(*n* = 19075)**		
Age at index — yr	67 ± 10	68 ± 13	0.622	0.005
Male sex — no. (%)	13344 (70)	13316 (70)	0.755	0.003
Body-mass index	32 ± 7	31 ± 8	0.105	0.017
Body surface area	2.1 ± .3	2.1 ± .4	0.938	0.001
Left Ventricular Ejection Fraction (LVEF)				
Available — no. (%)	5718 (30)	5662 (30)	0.531	0.006
LVEF (%)	46 ± 14	46 ± 16	0.456	0.014
LVEF ≥ 40 to 50% — no. (%)	2590 (14)	2629 (14)	0.190	0.014
LVEF < 40% — no. (%)	3128 (16)	3033 (16)	0.190	0.014
Persistent atrial fibrillation — no. (%)	12754 (67)	12976 (68)	0.015	0.025
Typical atrial flutter — no. (%)	3215 (17)	3254 (17)	0.595	0.005
Atypical atrial flutter — no. (%)	2006 (11)	2110 (11)	0.086	0.018
Heart rate — bpm	77 ± 20	77 ± 18	0.069	0.019
Cardiomyopathy — no. (%)	12020 (63)	11875 (62)	0.125	0.016
Dilated cardiomyopathy — no. (%)	3726 (20)	3673 (19)	0.415	0.008
Ischemic cardiomyopathy — no. (%)	3570 (19)	3637 (19)	0.381	0.009
Ischemic heart disease — no. (%)	12695 (67)	12664 (66)	0.737	0.003
Hypertensive diseases — no. (%)	16467 (86)	16597 (87)	0.050	0.020
COPD — no. (%)	3989 (21)	4020 (21)	0.697	0.004
Diabetes mellitus — no. (%)	7105 (37)	7074 (37)	0.743	0.003
Cerebral infarction — no. (%)	1673 (9)	1647 (9)	0.637	0.005
Presence of cardiac pacemaker — no. (%)	2425 (13)	2521 (13)	0.143	0.015
Presence of ICD — no. (%)	2728 (14)	2853 (15)	0.070	0.019
NT-pro BNP available — no. (%)	7088 (37)	7020 (37)	0.471	0.007
NT-pro BNP — pg/ml	3262 ± 6161	3925 ± 7277	0.001	0.098
Creatinine — mg/dl	1.3 ± 3	1.4 ± 2	0.020	0.024
Glomerular filtration rate (MDRD) — ml/min	64 ± 23	65 ± 28	0.027	0.023
Medication — no. (%)				
Beta blocker	17729 (93)	17785 (93)	0.258	0.012
Diuretics	15519 (81)	15549 (82)	0.693	0.004
RAS-inhibitor	18118 (95)	18096 (95)	0.610	0.005
ACE-inhibitor	8435 (44)	8562 (45)	0.191	0.013
Angiotensin II inhibitor	9683 (51)	9534 (50)	0.127	0.016
Sacubitril - valsartan	3929 (21)	3666 (19)	0.001	0.035
Spironolactone	6594 (35)	6481 (34)	0.223	0.012
SGLT-2 inhibitor	3819 (20)	3469 (18)	<0.001	0.047
Antiarrhythmics	16993 (89)	17039 (89)	0.448	0.008
Class I and III	16951 (89)	16993 (89)	0.492	0.007
Class III	11540 (60)	11634 (61)	0.324	0.010
Amiodarone	10069 (53)	10073 (53)	0.967	<0.001
Digitalis glycosides	4436 (23)	4464 (23)	0.735	0.003
Anticoagulants	18086 (95)	18220 (96)	0.001	0.033
Warfarin	3726 (20)	3914 (21)	0.016	0.025

*Plus-minus values are means +SD. Body mass index is calculated as kilograms per square meter, Body surface area is calculated per square meter, Glomerular filtration rate is calculated as volume rate per 1.73 square meter. *COPD* denotes Chronic obstructive pulmonary disease, *ICD* implantable cardioverter defibrillator, *RAS* renin-angiotensin system, *ACE* angiotensin-converting enzyme, *SGLT-2* sodium-glucose transport protein 2 and *SD* standard deviation.

### Primary endpoint, all-cause mortality, and hospitalizations

**Fig. 1:**
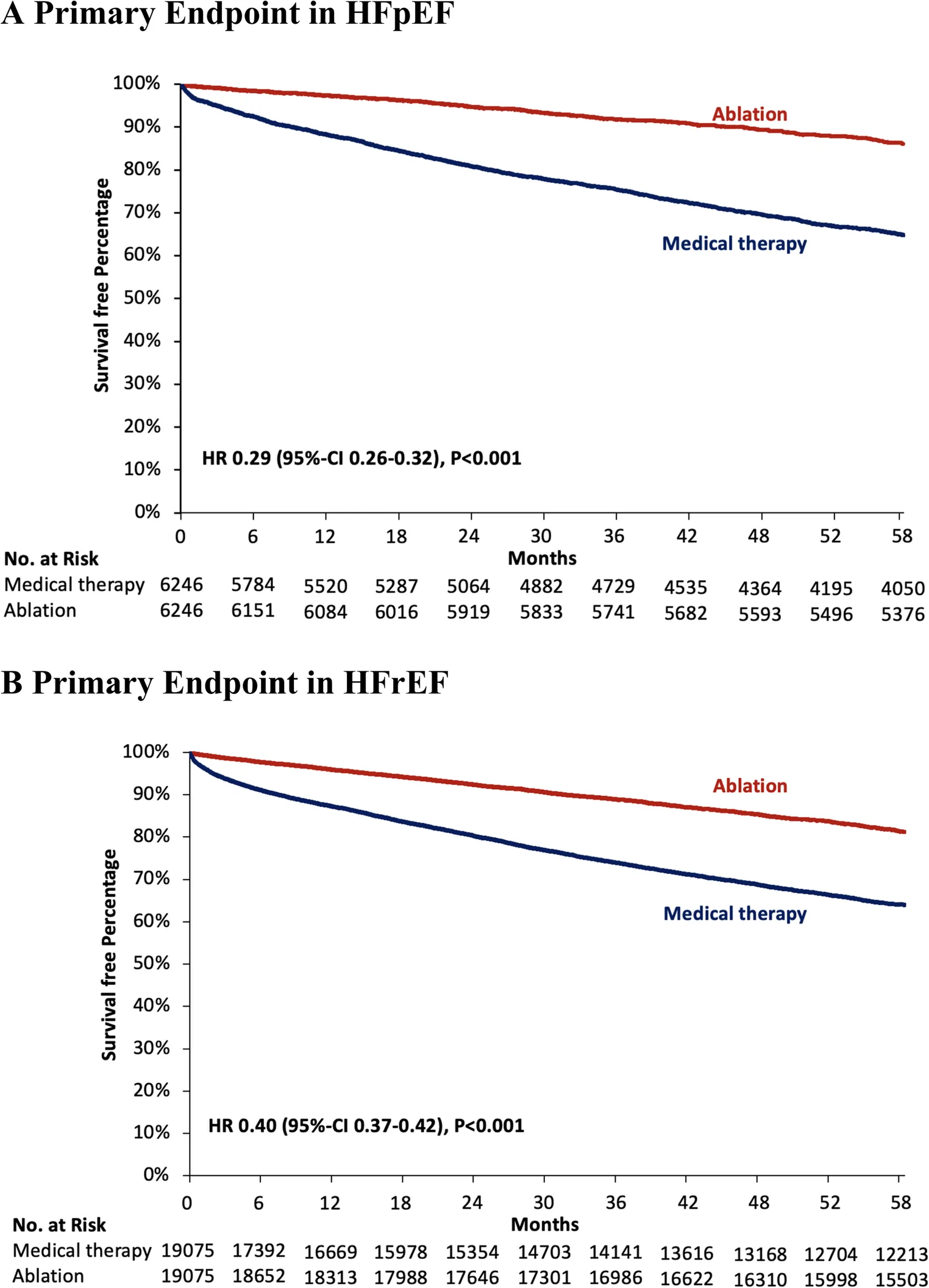
Incidence of primary endpoint events. Kaplan-Meier survival curve demonstrating decreased risk of primary Endpoint in patients with co-existing AF and HFpEF (**A**) and HFrEF (**B**). In the HFpEF cohort, the primary endpoint occurred in 379 patients (6.07%) after ablation compared to 1,777 patients (28.5%) in the medical therapy group. In HFrEF cohort, the primary endpoint occurred in 1,780 patients (9.33%) after ablation compared to 5,012 patients (26.3%) in medical therapy group

Over a mean follow-up of 973 ± 609 days in the HFpEF cohort and 926 ± 603 days in the HFrEF cohort, catheter ablation was associated with significantly fewer primary endpoint events compared with medical therapy (HFpEF: 379 vs. 1,777; HFrEF: 1,780 vs. 5,012) and lower all-cause mortality events (HFpEF: 378 vs. 1,776; HFrEF: 1,723 vs. 4,869). Catheter ablation reduced the risk of the primary endpoint and mortality by over 71% in the HFpEF and 60% in the HFrEF group (HFpEF: hazard ratio (HR) 0.29; HFrEF: HR 0.40; all *P* < 0.001) (Fig. 1). In both cohorts, the primary endpoint was primarily driven by mortality.

**Fig. 2:**
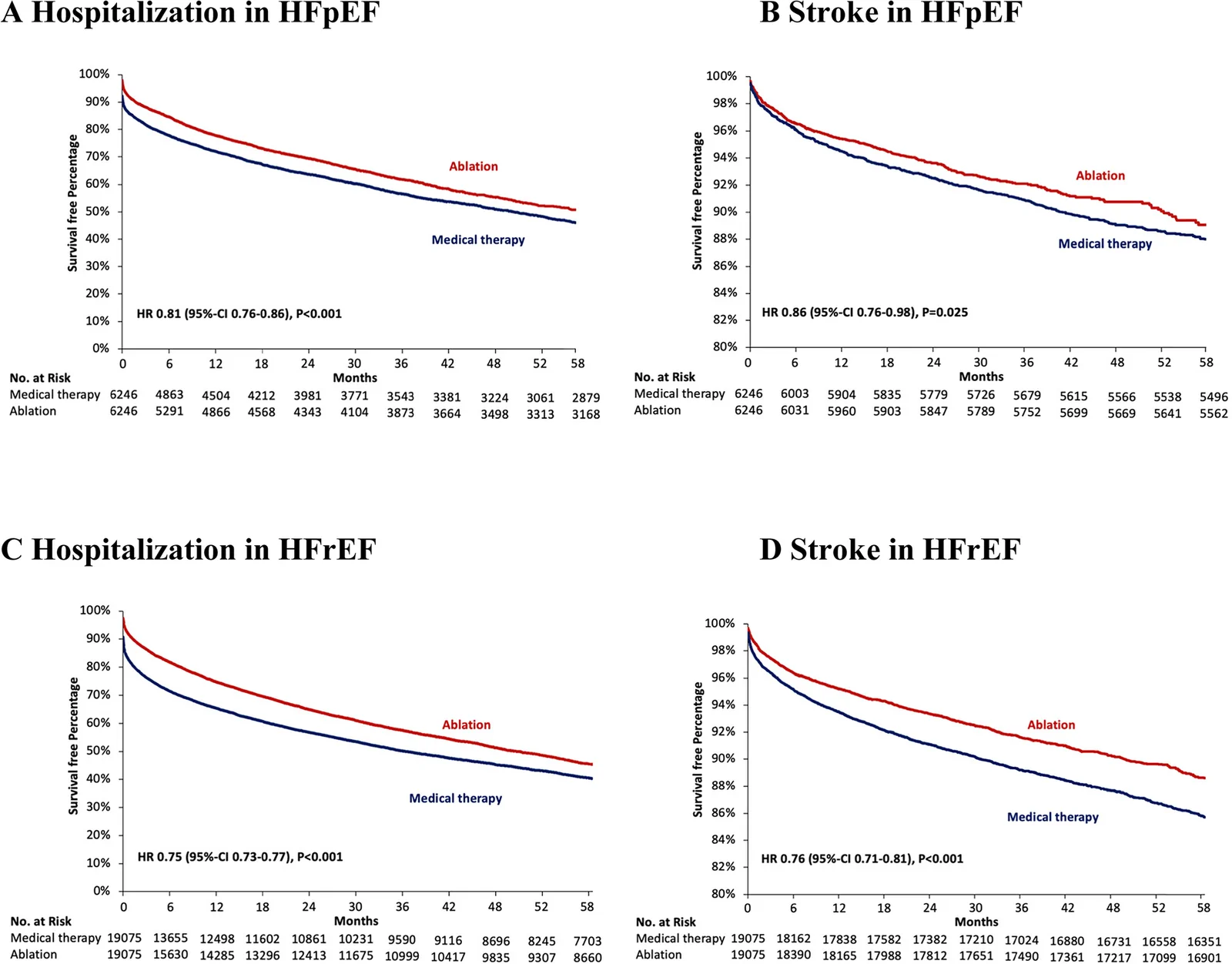
Incidence of hospitalization and stroke events. Kaplan-Meier survival curve demonstrating decreased risk of hospitalization and stroke events patients with co-existing AF and HFpEF (**A**-**B**) and HFrEF (**C**-**D**). In the HFpEF cohort, the hospitalization rates were 30.5% (1,903 patients) after ablation versus 44.3% (2,767 patients) in the medical therapy group, while stroke rates were 6.34% (396 patients) versus 9.03% (564 patients) in medical therapy group. In HFrEF cohort, the hospitalization rates were 36.0% (6,865 patients) after ablation versus 47.6% (9,077 patients) in the medical therapy group, and stroke rates were 6.94% (1,323 patients) versus 9.89% (1,886 patients) in the medical therapy group

Ablation was also associated with significantly lower rates of hospitalization rates in both cohorts (HFpEF: HR 0.81, 95%-CI 0.76–0.86; HFrEF: HR 0.75, 95%-CI 0.73–0.77, all *P* < 0.001) (Fig. 2).

### Cerebrovascular events and antiarrhythmic therapy

Catheter ablation was associated with a lower incidence of cerebrovascular events (including TIA and stroke) in the HFrEF (HR 0.80, 95%-CI 0.75–0.85, *p* < 0.001), and the HFpEF group (HR 0.89, 95%-CI 0.80-1.00, *p* < 0.043). However, when analyzed separately, stroke rates were significantly reduced in both groups following catheter ablation (HFpEF: HR 0.86, 95%-CI 0.76–0.98, *p* = 0.025; HFrEF: HR 0.76, 95%-CI 0.71–0.81, *p* < 0.001) (Fig. 2).

**Fig. 3:**
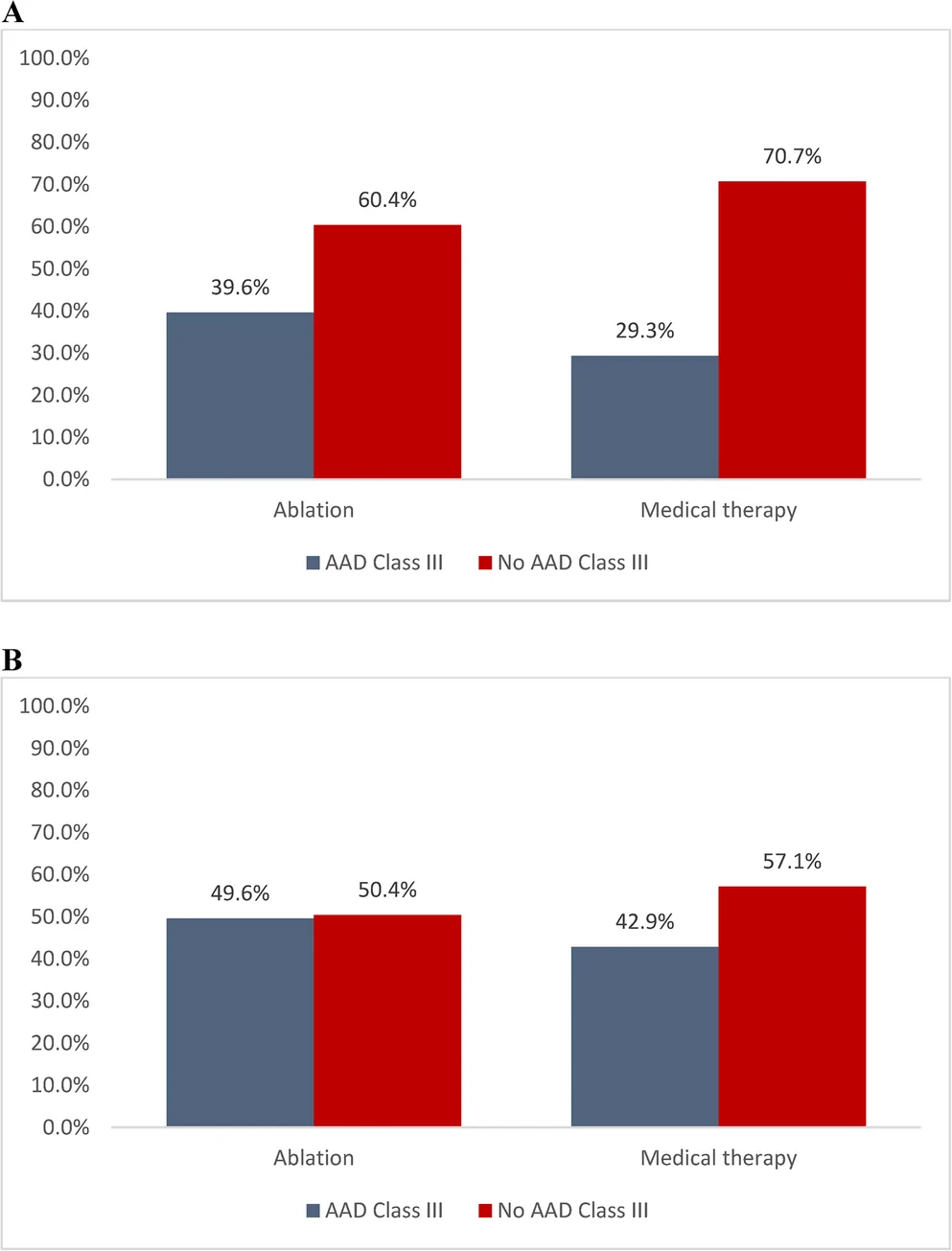
Incidence of antiarrhythmic drug use. Class III antiarrhythmics use in patients with coexisting AF and HFpEF is shown in panel **A** and for AF and HFrEF in panel **B**. In the HFpEF cohort, 9,457 patients (49.6%) were taking class III antiarrhythmics after ablation compared to 8,175 patients (50.4%) in the medical therapy group. In HFrEF, the primary endpoint occurred in 1,780 patients (9.33%) after ablation compared to 5,012 patients (26.3%) in medical therapy group. Analyses were conducted using time-to-first event analysis without including a blanking period following ablation

Despite higher cardioversion rates in the ablation cohorts (HFpEF: HR 2.05, 95%-CI 1.90–2.21; HFrEF: HR 2.01, 95%-CI 1.92–2.11; all *p* < 0.001), AAD use increased in the HFpEF and HFrEF group (HFpEF: HR 1.37, 95%-CI 1.31–1.43; HFrEF: HR 1.08, 95%-CI 1.06–1.11, all *p* < 0.001). Subsequent analysis demonstrated increased use of class III AADs after ablation in both heart failure groups indicating the absence of a blanking period (HFpEF: HR 1.73, 95%-CI 1.63–1.84; HFrEF: HR 1.38, 95%-CI 1.38–1.42; all *p* < 0.001) (Fig. 3).

## Discussion

In this real-world, propensity-matched cohort study of HF patients with concomitant AF, catheter ablation was associated with a significant reduction in primary composite endpoint of all-cause mortality, left-ventricular-assist-device implantation and heart transplantation compared with medical therapy alone. Additionally, ablation was linked to a lower incidence of all-cause mortality by over 71% in the HFpEF and 60% in the HFrEF group, with mortality being the primary contributor to the composite endpoint in both cohorts. Moreover, stroke incidences were reduced by 11% in HFpEF vs. 20% in HFrEF and hospitalizations by 14% in HFpEF vs. 24% in HFrEF, underscoring its potential prognostic benefit across the spectrum of HF phenotypes.

### Mortality benefit

Several major randomized controlled trials, including CASTLE-AF, CABANA, and CASTLE-HTx have demonstrated that catheter ablation of AF in patients with HF, particularly HFrEF, significantly reduces mortality, HF progression, and hospitalization. However, the benefit of AF ablation in patients with HFpEF remains less clearly defined. Whereas a metanalysis and smaller cohort studies have reported comparable outcomes between HFpEF and HFrEF following AF ablation, others found no significant reduction in mortality or HF progression among patients with HFpEF [1, 2, 6–12]. The current analysis demonstrates that mortality was the main driver within the composite endpoint, particularly in HFpEF, with non-fatal events contributing only two cases (0.09% of events) in HFpEF patients and 200 cases (2.94% of events) in HFrEF patients. The magnitude of the observed treatment effect, particularly within the HFpEF cohort, exceeds findings from randomized clinical trials and requires careful interpretation [1, 2, 4, 13, 14]. Despite comprehensive propensity score matching, residual confounding and selection bias cannot be excluded. In addition, the early separation of Kaplan-Meier curves suggests the possibility for unmeasured confounding factors and an overestimation of the treatment effect. Therefore, these findings should be considered hypothesis-generating and require validation through prospective randomized trials; however, they indicate an important knowledge gap regarding the efficacy of AF ablation in this population.

### Reduction of cerebrovascular events

Our findings suggest that the pathophysiological differences between HFpEF and HFrEF may not substantially influence the prognostic benefit of AF ablation, particularly with respect to hard clinical outcomes such as mortality, stroke, and hospitalization. The observed reduction in stroke risk aligns with recent data from OCEAN trial, which demonstrated low stroke risk at three-year follow up among patients who remained free of AF recurrence within a year of ablation regardless of antithrombotic regimen after one-year of ablation (Comparing Aspirin to Rivaroxaban). However, the proportion of HF patients in OCEAN remains unclear [15]. Although AF burden data and longitudinal anticoagulation have not been evaluated in our study, there might be a potential association between successful rhythm control or lower AF burden and a lower stroke risk. Recent meta-analyses of randomized controlled trials support our findings and report a stroke reduction of 30 to 40% [16, 17]. Nonetheless, the observed association between ablation and lower stroke incidence should be interpreted cautiously, as residual confounding cannot be excluded.

### Antiarrhythmic therapy

Catheter ablation did not result in significant reductions in the need for cardioversion or AAD use in the HFpEF or HFrEF cohort, suggesting that adjunctive pharmacotherapy may still be needed for maintaining sinus rhythm after ablation. In the present analysis, AAD exposure indicates prescriptions recorded during the follow-up period rather than time-varying AAD use due to analytical limitations. Therefore, the higher AAD use should not be interpreted as treatment failure but may reflect temporary rhythm-stabilization strategies or treatment of early recurrences after ablation. Class III AADs were used frequently in both HF groups, and the continued reliance on AADs after ablation in HFpEF patients may reflect the higher propensity for AF recurrence in this population. Moreover, this pattern may underscore the clinical importance of maintaining sinus rhythm in HFpEF, as these patients often experience a greater symptomatic burden when AF recurs, prompting more aggressive rhythm-control strategies [18, 19]. By reducing AF burden, catheter ablation can mitigate the progression of left atrial myopathy, limit left atrial dilation, and improve left atrial strain and overall hemodynamics, which may translate into reduced HF progression, improved symptoms, and mortality [20].

### Clinical relevance

Given the increasing prevalence of HFpEF in the aging population and the well-established adverse impact of AF on this patient population, our findings hold substantial clinical relevance. AF exacerbates HF symptoms, increases hospitalization rates, and worsens long-term prognosis. Demonstrating that catheter ablation provides comparable benefits in HFpEF as in HFrEF supports broader application of AF ablation and emphasizes the value of early rhythm-control strategies in HFpEF patients. CABA-HFPEF and CASTLE-HFpEF (ClinicalTrials.gov number: NCT 07254455) trials are currently evaluating the role of catheter ablation in the HFpEF population [21].

## Limitations

This is a retrospective observational study, and thus subject to selection bias and unmeasured confounders, which limit the ability to establish direct causality. Despite the alignment of follow-up through predefined index events (AF diagnosis vs. ablation procedure) and the application of propensity score matching to balance baseline characteristics, residual bias associated with retrospective observational analyses may remain. Patients undergoing ablation have to survive until the procedure is conducted, potentially introducing a survival advantage which is independent of the treatment effect. Consequently, the applied methodology mitigates, yet does not eliminate, this bias, necessitating careful interpretation of the results. Variability in disease coding and data recording across participating institutions may have introduced additional heterogeneity. In addition, differences in healthcare access and treatment availability among patient may have influenced outcomes. Ablation and non-ablation patients were derived from multiple participating healthcare organizations within the TriNetX network, with partially different institutions contributing to both cohorts. Although the federated database structure allows longitudinal tracking of patients across institutions, potential referral patterns between centers and differences in local treatment strategies may have introduced additional selection bias. Furthermore, HF related hospitalizations were not analyzed separately, as such events may be inconsistently coded across institutions. The primary composite endpoint was primarily driven by all-cause mortality, particularly in the HFpEF cohort, where non-fatal events such as advanced HF therapies were infrequent. Therefore, the composite endpoint should be interpreted with caution, as it may not fully capture the spectrum of clinically relevant outcomes in this population.

Detailed procedural information including the ablation technique, operator expertise, and ablation energy source was unavailable, which may affect long-term outcomes. Furthermore, longitudinal data on antiarrhythmic therapy, AF burden, or echocardiographic parameters, such as LVEF or left atrial strain, and serial laboratory parameters, such as NT-pro BNP, were not consistently available, limiting mechanistic interpretation. Periprocedural anticoagulation management and antiarrhythmic strategies for early AF or atrial flutter recurrence after ablation are both part of routine clinical practice but were not available within the TriNetX database and therefore could not be analyzed. Due to the lack of a specific ICD code for HFmrEF and limited availability of LVEF measurements for cohort classification, the HFmrEF patients have been included in the HFrEF cohort. Individual stroke risks by including CHA₂DS₂-VASc score were not available in the database and therefore not included in the analysis. Moreover, cardiac resynchronization therapy (CRT) as well as conduction system pacing devices could not be consistently differentiated from pacemakers or implantable cardioverter-defibrillators within the existing TriNetX coding framework and were consequently not examined as a distinct variable.

Furthermore, current ESC and AHA/ACC/HFSA guidelines recommend the use of SGLT2 inhibitors and mineralocorticoid receptor antagonists in patients with both HFpEF and HFrEF. In our study, these therapies were represented in only a minority, particularly within the HFpEF cohort [22, 23]. This may have influenced the HF progression; however, the limited use was comparable between the catheter ablation and medical therapy groups in the HFpEF cohort.

Nonetheless, propensity score matching was applied to minimize baseline imbalances and improve the validity of our findings. Despite these limitations, the use of a large, multicenter real-world dataset enhances the validity and generalizability of our results in patients with HF with AF.

## Conclusion

In this large, retrospective, real-world analysis, **catheter ablation** was associated with a reduction in the risks of **death**, **advanced heart failure therapies**, **stroke**, and **hospitalization** among patients with **HFpEF** and **HFrEF** and coexisting **atrial fibrillation**. These findings suggest that catheter ablation may yield favorable outcomes across various HF phenotypes; however, prospective randomized studies are needed to validate these observations.

## Supplementary Information

Below is the link to the electronic supplementary material.

Supplementary Material 1 (DOOCX 1.50 MB)

## Data Availability

This study utilized data from TriNetX, a global clinical research network that aggregates de-identified electronic medical records data from participating health care organizations. The TriNetX database integrates data including patients’ demographics, diagnoses, procedures, medications, and laboratory results. All data provided through TriNetX network are de-identified and aggregated, ensuring compliance with the Health Insurance Portability and Accountability Act and other applicable US government regulations. Further information about the TriNetX network is publicly available at https://trinetx.com/data-sets-analytics/.

## Abbreviations

AAD: Antiarrhythmic drug
AF: Atrial fibrillation
CI: Confidence interval
HF: Heart failure
HFpEF: Heart failure with preserved ejection fraction
HFrEF: Heart failure with reduced ejection fraction
HR: Hazard ratio
ICD: International classification of diseases
LVEF: Left ventricular ejection fraction
SGLT2: Sodium-glucose co-transporter 2
