# Supplementary material for: Catheter ablation of atrial fibrillation in patients with heart failure - preserved and reduced ejection fraction - real world evidence
Source: J Interv Card Electrophysiol. 2026 May 26;69(8):2499–508. doi: 10.1007/s10840-026-02349-z (PMC13739959; doi:10.1007/s10840-026-02349-z)
Supplement: Supplementary file 1 [file 10840_2026_2349_MOESM1_ESM.docx]

## Supplemental Material

**Supplementary Table 1.** ICD-10-CM codes used for diagnosis of comorbidities

| **Diagnosis** | **ICD-10-CM Code** |
| --- | --- |
| Diastolic (congestive) heart failure | I50.3 |
| Systolic (congestive) heart failure | I50.2 |
| Combines diastolic and systolic heart failure | I50.4 |
| Atrial Fibrillation | I48.0 |
|  | I48.1 |
|  | I48.2 |
|  | I48.91 |
| Hypertensive diseases | I10-I1A |
| Diabetes mellitus | E08-E13 |
| Other chronic obstructive pulmonary disease | J44 |
| Cerebral infarction | I63 |
| Transient Ischemic Attack | G45 |
| Cardiomyopathy | I42 |
| Dilated cardiomyopathy | I42.0 |
| Ischemic cardiomyopathy | I25.5 |
| Ischemic heart disease | I20-I25 |
| Typical atrial flutter | I48.3 |
| Atypical atrial flutter | I48.4 |
| Presence of cardiac pacemaker | Z95.0 |
| Presence of automatic (implantable) cardiac defibrillator | Z95.810 |

**Supplementary Table 2.** Variables included in propensity score

| **Demographics** | Age at Index |
| --- | --- |
|  | Sex |
| **Diagnosis** | Hypertensive diseases |
|  | Diabetes mellitus |
|  | Other chronic obstructive pulmonary disease |
|  | Type of atrial Fibrillation |
|  | Diastolic (congestive) heart failure |
|  | Cerebral infarction |
|  | Cardiomyopathy |
|  | Dilated cardiomyopathy |
|  | Ischemic cardiomyopathy |
|  | Ischemic heart disease |
|  | Typical atrial flutter |
|  | Atypical atrial flutter |
|  | Presence of cardiac pacemaker |
|  | Presence of automatic (implantable) cardiac defibrillator |
| **Medications** | Cardiovascular medications |
|  | Beta blocker |
|  | Diuretics |
|  | ACE-inhibitor |
|  | Angiotensin II inhibitor |
|  | Spironolactone |
|  | Sacubitril |
|  | Sodium-glucose co-transporter 2 (SGLT2) inhibitors |
|  | Antiarrhthythmics |
|  | Class I and III |
|  | Class III |
|  | Amiodarone |
|  | Sotalol |
|  | Flecainide |
|  | Digitalis glycosides |
|  | Anticoagulants |
|  | Warfarin |
| **Laboratory parameters** | Body-mass index |
|  | Body surface area |
|  | Heart rate — bpm |
|  | Creatinine [Mass/volume] in Serum, Plasma or Blood |
|  | Glomerular filtration rate/1.73 sq M.predicted [Volume Rate/Area] in Serum, Plasma or Blood by Creatinine-based formula (MDRD) |
|  | Natriuretic peptide.B prohormone N-Terminal [Mass/volume] in Serum, Plasma or Blood |
|  | Left Ventricular Ejection Fraction (LVEF) (%) |

**Supplementary Table 3.** Baseline characteristics before propensity score matching- extended*

*Plus–minus values are means ±SD. Body mass index is calculated as kilograms per square meter, Body surface area is calculated per square meter, Glomerular filtration rate is calculated as volume rate per 1.73 square meter. COPD denotes Chronic obstructive pulmonary disease, ICD implantable cardioverter defibrillator, ACE angiotensin-converting enzyme, SGLT-2 sodium-glucose transport protein 2, GFR glomerular filtration rate, LVEF left ventricular ejection fraction and SD standard deviation.

**Supplementary Table 4**. Baseline characteristics after propensity score matching – extended*

*Plus–minus values are means ±SD. Body mass index is calculated as kilograms per square meter, Body surface area is calculated per square meter, Glomerular filtration rate is calculated as volume rate per 1.73 square meter. COPD denotes Chronic obstructive pulmonary disease, ICD implantable cardioverter defibrillator, ACE angiotensin-converting enzyme, SGLT-2 sodium-glucose transport protein 2, GFR glomerular filtration rate, LVEF left ventricular ejection fraction and SD standard deviation.
